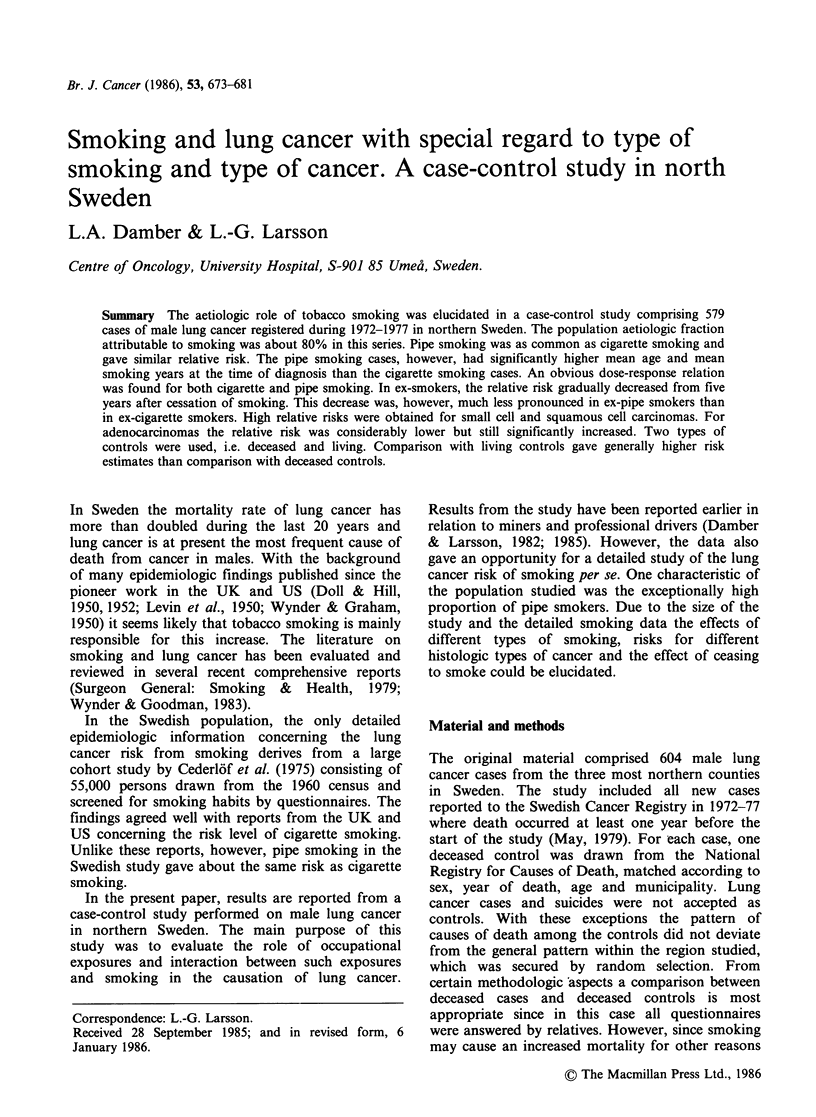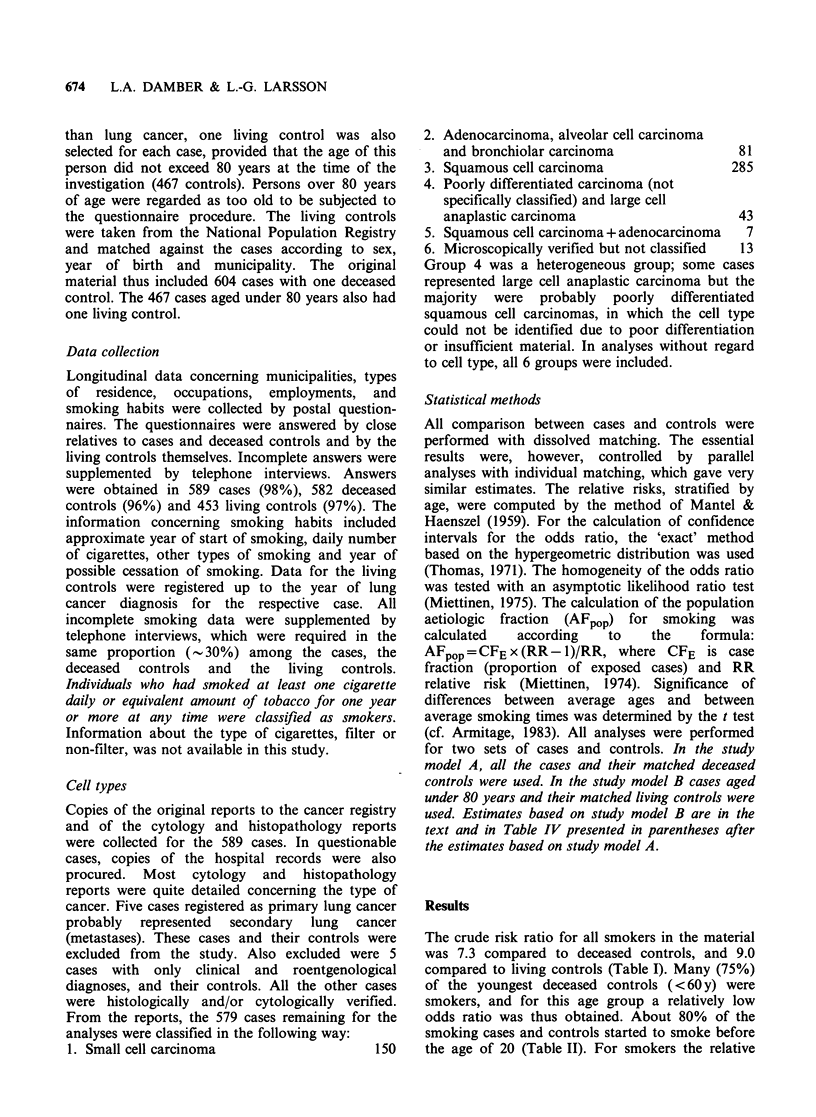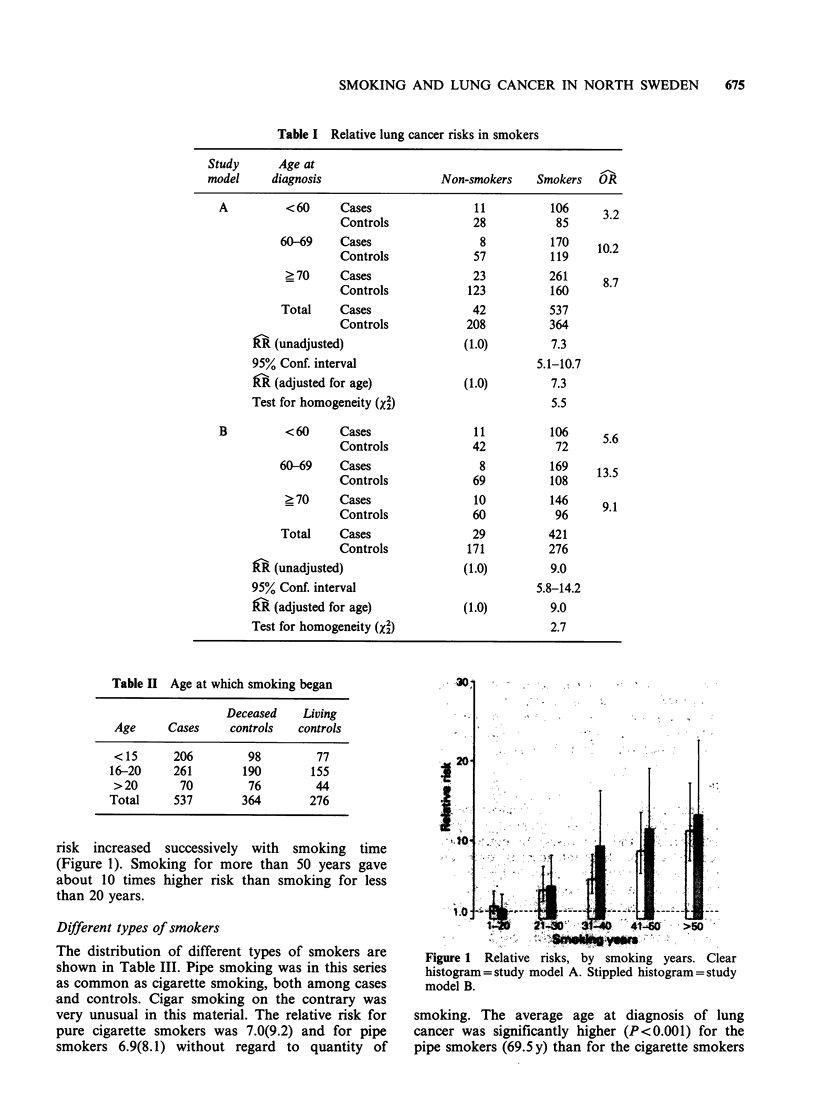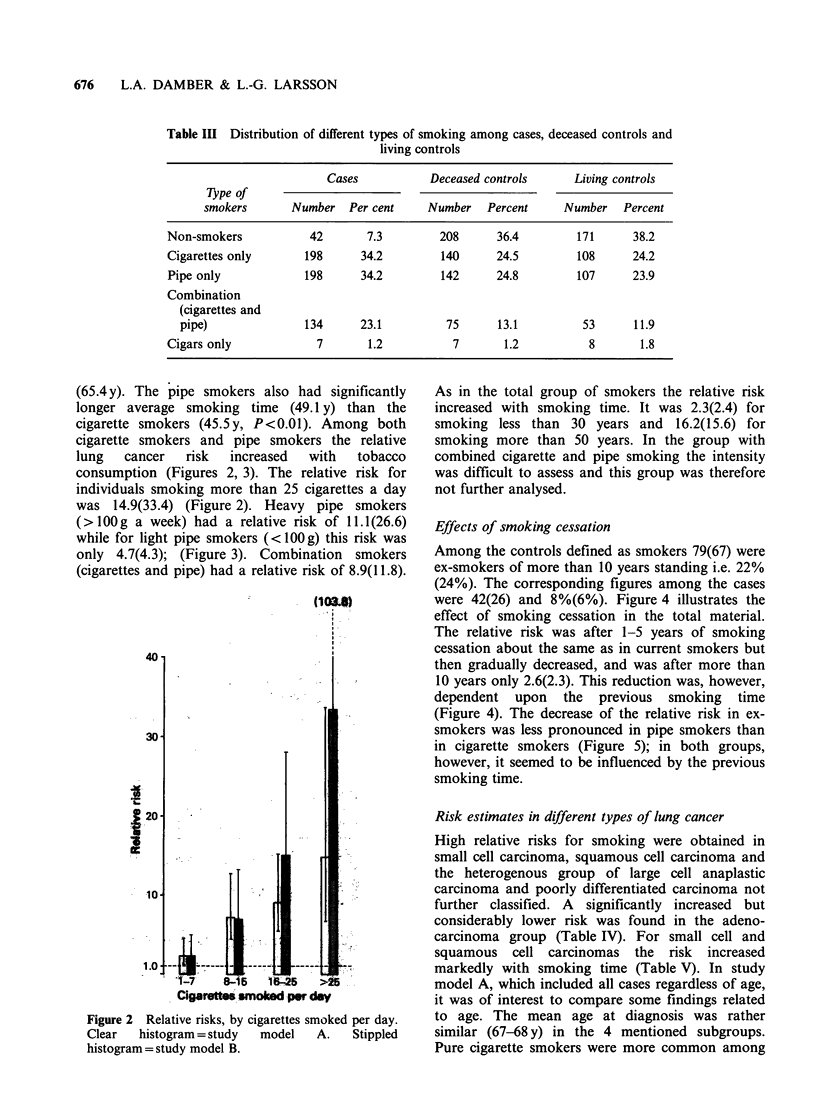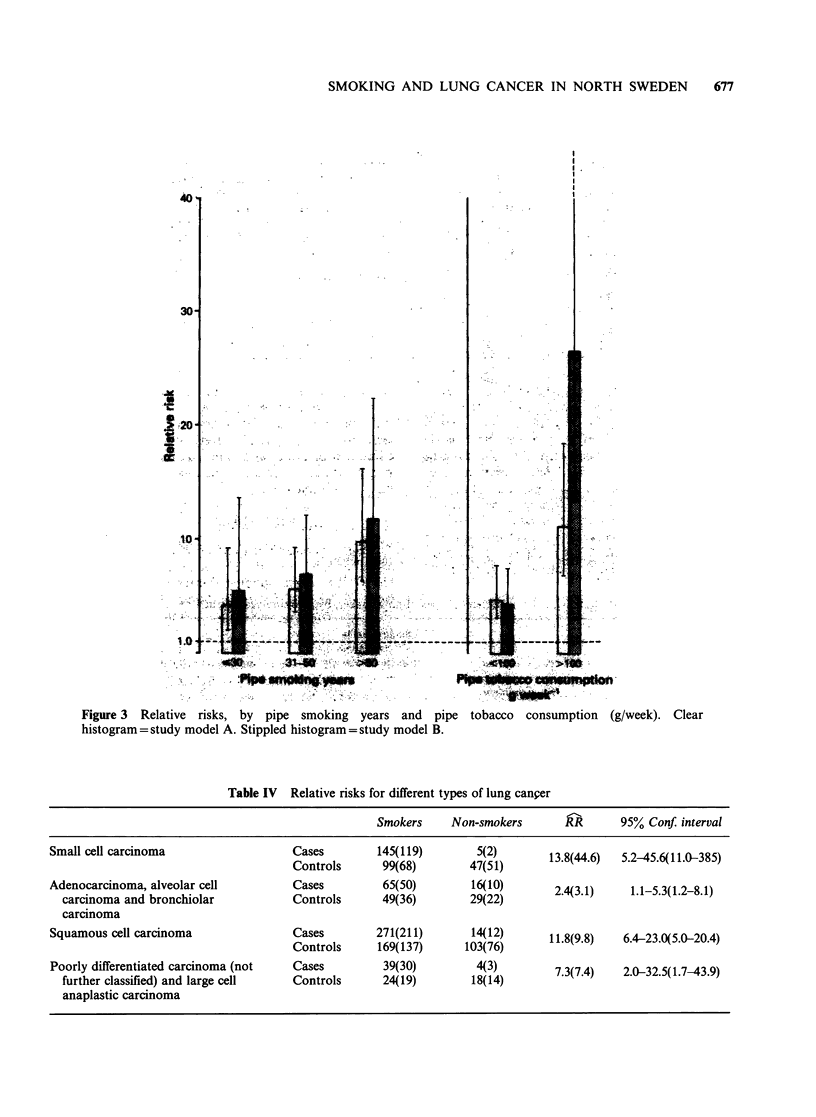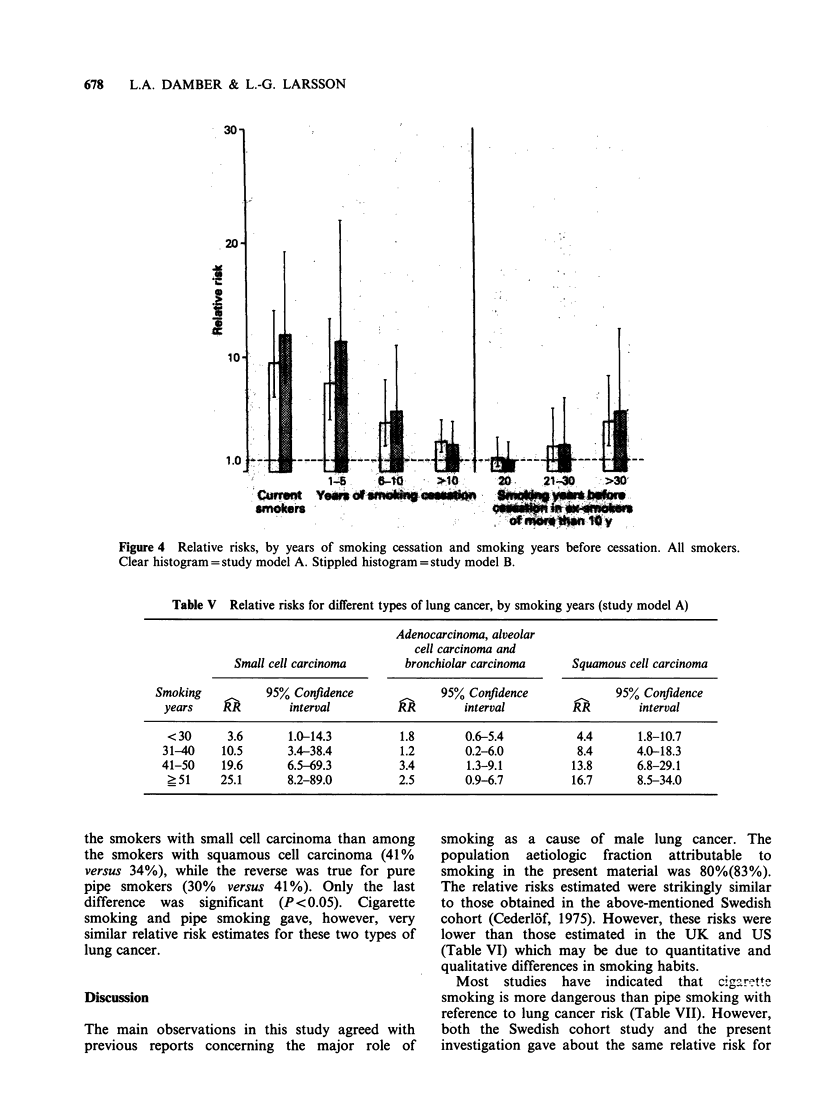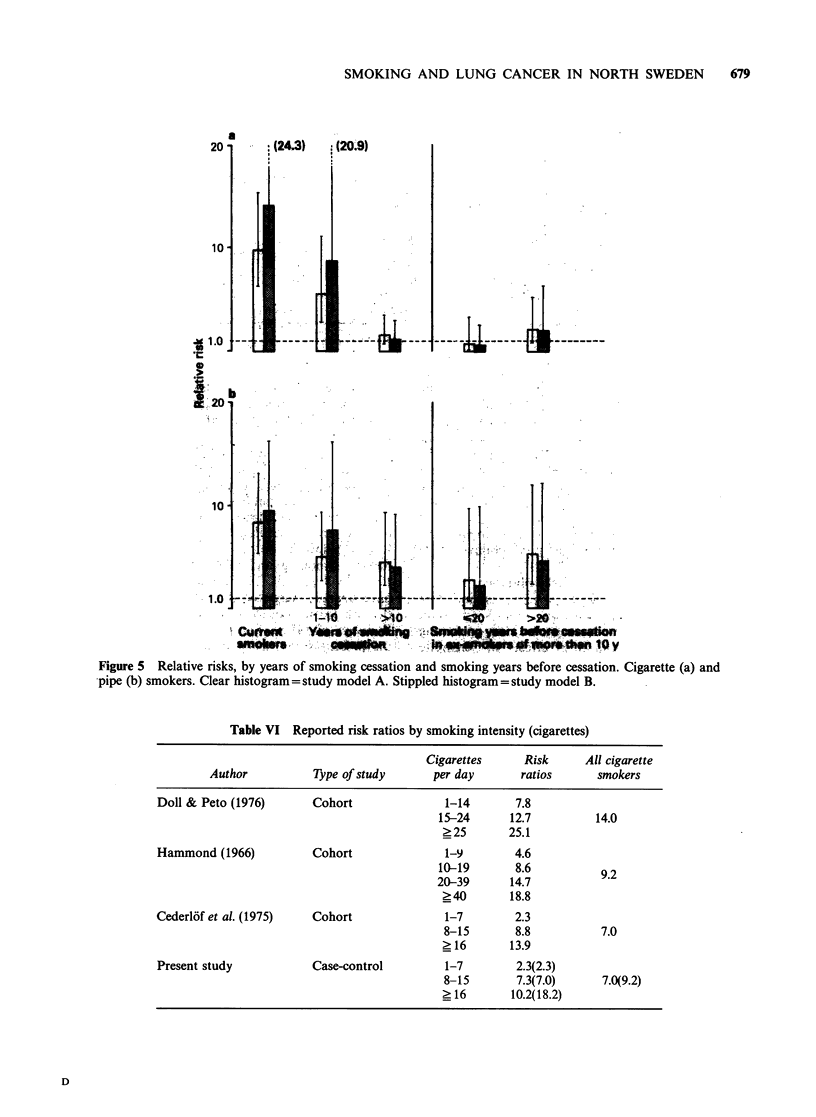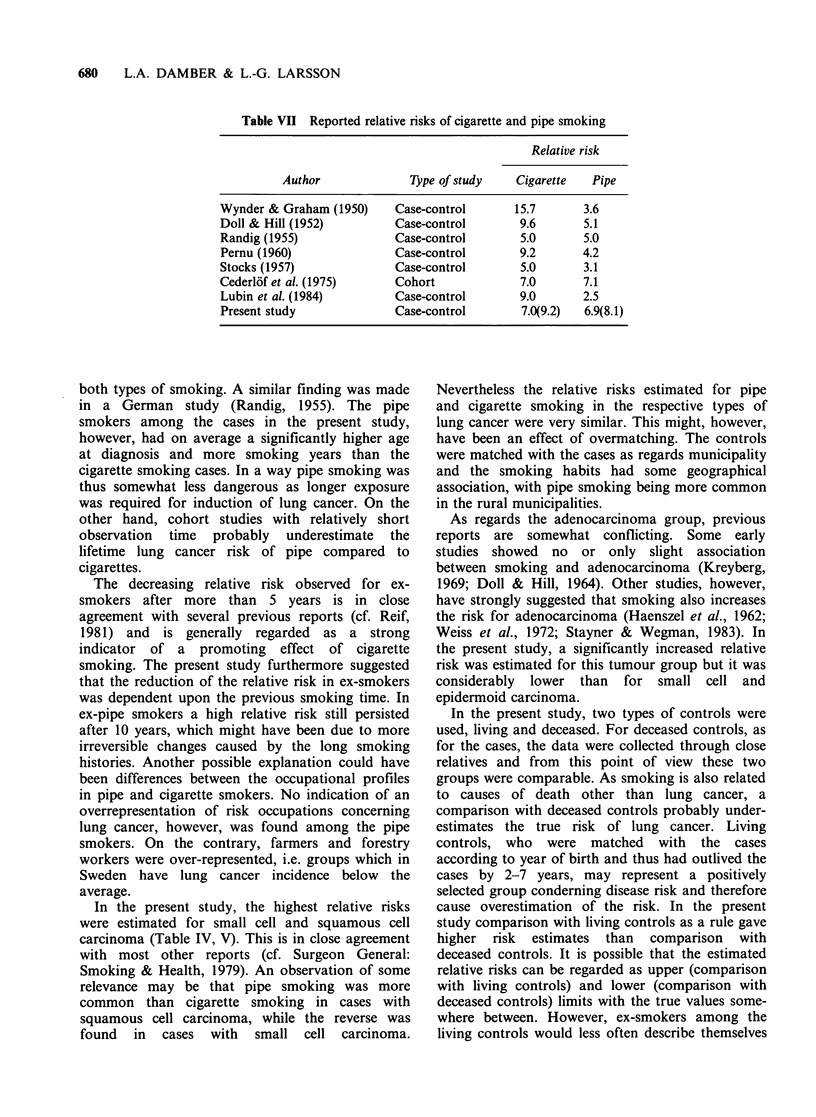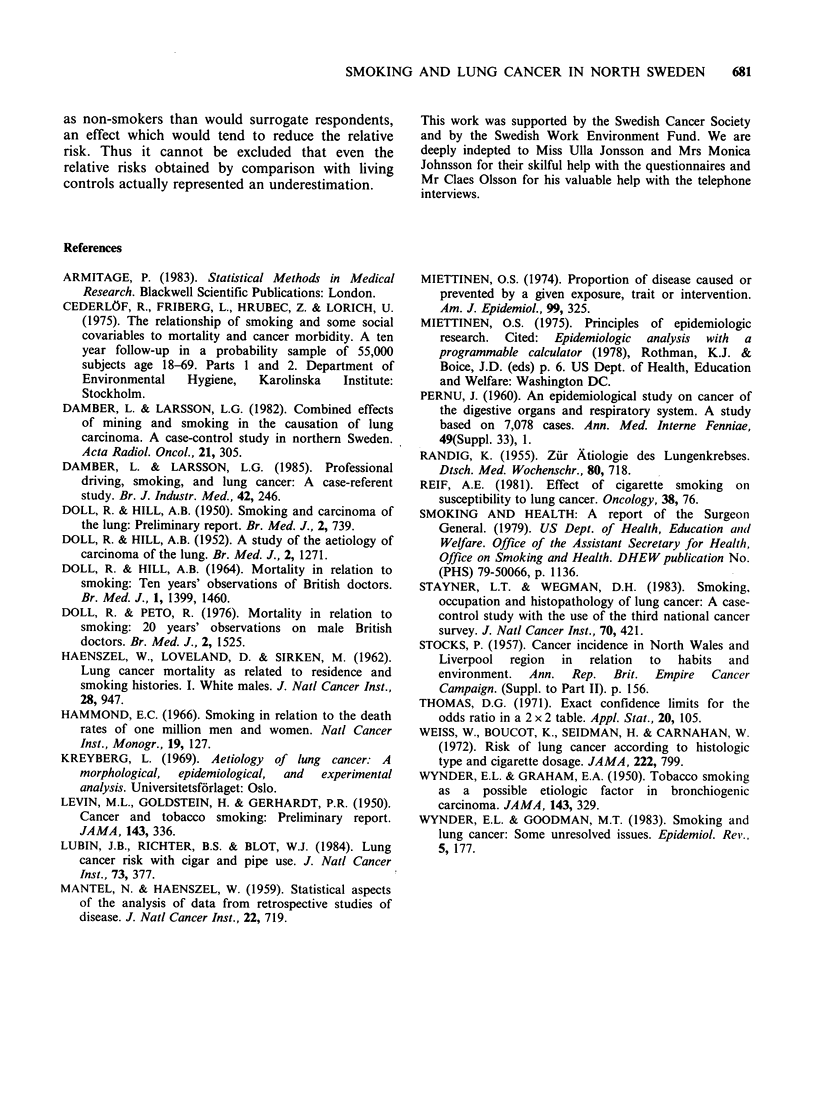# Smoking and lung cancer with special regard to type of smoking and type of cancer. A case-control study in north Sweden.

**DOI:** 10.1038/bjc.1986.111

**Published:** 1986-05

**Authors:** L. A. Damber, L. G. Larsson

## Abstract

The aetiologic role of tobacco smoking was elucidated in a case-control study comprising 579 cases of male lung cancer registered during 1972-1977 in northern Sweden. The population aetiologic fraction attributable to smoking was about 80% in this series. Pipe smoking was as common as cigarette smoking and gave similar relative risk. The pipe smoking cases, however, had significantly higher mean age and mean smoking years at the time of diagnosis than the cigarette smoking cases. An obvious dose-response relation was found for both cigarette and pipe smoking. In ex-smokers, the relative risk gradually decreased from five years after cessation of smoking. This decrease was, however, much less pronounced in ex-pipe smokers than in ex-cigarette smokers. High relative risks were obtained for small cell and squamous cell carcinomas. For adenocarcinomas the relative risk was considerably lower but still significantly increased. Two types of controls were used, i.e. decreased and living. Comparison with living controls gave generally higher risk estimates than comparison with deceased controls.


*Br. J. Cancer* (1986), **53**, 673–681

### Smoking and lung cancer with special regard to type of smoking and type of cancer. A case-control study in north Sweden

L.A. Damber & L.-G. Larsson

*Centre of Oncology, University Hospital, S-901 85 Umeå, Sweden.*

**Summary** The aetiologic role of tobacco smoking was elucidated in a case-control study comprising 579 cases of male lung cancer registered during 1972–1977 in northern Sweden. The population aetiologic fraction attributable to smoking was about 80% in this series. Pipe smoking was as common as cigarette smoking and gave similar relative risk. The pipe smoking cases, however, had significantly higher mean age and mean smoking years at the time of diagnosis than the cigarette smoking cases. An obvious dose-response relation was found for both cigarette and pipe smoking. In ex-smokers, the relative risk gradually decreased from five years after cessation of smoking. This decrease was, however, much less pronounced in ex-pipe smokers than in ex-cigarette smokers. High relative risks were obtained for small cell and squamous cell carcinomas. For adenocarcinomas the relative risk was considerably lower but still significantly increased. Two types of controls were used, i.e. deceased and living. Comparison with living controls gave generally higher risk estimates than comparison with deceased controls.

In Sweden the mortality rate of lung cancer has more than doubled during the last 20 years and lung cancer is at present the most frequent cause of death from cancer in males. With the background of many epidemiologic findings published since the pioneer work in the UK and US (Doll & Hill, 1950, 1952; Levin *et al.*, 1950; Wynder & Graham, 1950) it seems likely that tobacco smoking is mainly responsible for this increase. The literature on smoking and lung cancer has been evaluated and reviewed in several recent comprehensive reports (Surgeon General: Smoking & Health, 1979; Wynder & Goodman, 1983).

In the Swedish population, the only detailed epidemiologic information concerning the lung cancer risk from smoking derives from a large cohort study by Cederlöf *et al.* (1975) consisting of 55,000 persons drawn from the 1960 census and screened for smoking habits by questionnaires. The findings agreed well with reports from the UK and US concerning the risk level of cigarette smoking. Unlike these reports, however, pipe smoking in the Swedish study gave about the same risk as cigarette smoking.

In the present paper, results are reported from a case-control study performed on male lung cancer in northern Sweden. The main purpose of this study was to evaluate the role of occupational exposures and interaction between such exposures and smoking in the causation of lung cancer. Results from the study have been reported earlier in relation to miners and professional drivers (Damber & Larsson, 1982; 1985). However, the data also gave an opportunity for a detailed study of the lung cancer risk of smoking *per se*. One characteristic of the population studied was the exceptionally high proportion of pipe smokers. Due to the size of the study and the detailed smoking data the effects of different types of smoking, risks for different histologic types of cancer and the effect of ceasing to smoke could be elucidated.

Correspondence: L.-G. Larsson.
Received 28 September 1985; and in revised form, 6 January 1986.

### Material and methods

The original material comprised 604 male lung cancer cases from the three most northern counties in Sweden. The study included all new cases reported to the Swedish Cancer Registry in 1972–77 where death occurred at least one year before the start of the study (May, 1979). For each case, one deceased control was drawn from the National Registry for Causes of Death, matched according to sex, year of death, age and municipality. Lung cancer cases and suicides were not accepted as controls. With these exceptions the pattern of causes of death among the controls did not deviate from the general pattern within the region studied, which was secured by random selection. From certain methodologic aspects a comparison between deceased cases and deceased controls is most appropriate since in this case all questionnaires were answered by relatives. However, since smoking may cause an increased mortality for other reasons

© The Macmillan Press Ltd., 1986

than lung cancer, one living control was also selected for each case, provided that the age of this person did not exceed 80 years at the time of the investigation (467 controls). Persons over 80 years of age were regarded as too old to be subjected to the questionnaire procedure. The living controls were taken from the National Population Registry and matched against the cases according to sex, year of birth and municipality. The original material thus included 604 cases with one deceased control. The 467 cases aged under 80 years also had one living control.

*Data collection*

Longitudinal data concerning municipalities, types of residence, occupations, employments, and smoking habits were collected by postal questionnaires. The questionnaires were answered by close relatives to cases and deceased controls and by the living controls themselves. Incomplete answers were supplemented by telephone interviews. Answers were obtained in 589 cases (98%), 582 deceased controls (96%) and 453 living controls (97%). The information concerning smoking habits included approximate year of start of smoking, daily number of cigarettes, other types of smoking and year of possible cessation of smoking. Data for the living controls were registered up to the year of lung cancer diagnosis for the respective case. All incomplete smoking data were supplemented by telephone interviews, which were required in the same proportion (~30%) among the cases, the deceased controls and the living controls. *Individuals who had smoked at least one cigarette daily or equivalent amount of tobacco for one year or more at any time were classified as smokers.* Information about the type of cigarettes, filter or non-filter, was not available in this study.

*Cell types*

Copies of the original reports to the cancer registry and of the cytology and histopathology reports were collected for the 589 cases. In questionable cases, copies of the hospital records were also procured. Most cytology and histopathology reports were quite detailed concerning the type of cancer. Five cases registered as primary lung cancer probably represented secondary lung cancer (metastases). These cases and their controls were excluded from the study. Also excluded were 5 cases with only clinical and roentgenological diagnoses, and their controls. All the other cases were histologically and/or cytologically verified. From the reports, the 579 cases remaining for the analyses were classified in the following way:

1. Small cell carcinoma 150
2. Adenocarcinoma, alveolar cell carcinoma and bronchiolar carcinoma 81
3. Squamous cell carcinoma 285
4. Poorly differentiated carcinoma (not specifically classified) and large cell anaplastic carcinoma 43
5. Squamous cell carcinoma + adenocarcinoma 7
6. Microscopically verified but not classified 13

Group 4 was a heterogeneous group; some cases represented large cell anaplastic carcinoma but the majority were probably poorly differentiated squamous cell carcinomas, in which the cell type could not be identified due to poor differentiation or insufficient material. In analyses without regard to cell type, all 6 groups were included.

*Statistical methods*

All comparison between cases and controls were performed with dissolved matching. The essential results were, however, controlled by parallel analyses with individual matching, which gave very similar estimates. The relative risks, stratified by age, were computed by the method of Mantel & Haenszel (1959). For the calculation of confidence intervals for the odds ratio, the 'exact' method based on the hypergeometric distribution was used (Thomas, 1971). The homogeneity of the odds ratio was tested with an asymptotic likelihood ratio test (Miettinen, 1975). The calculation of the population aetiologic fraction ($AF_{pop}$) for smoking was calculated according to the formula: $AF_{pop} = CF_E \times (RR - 1)/RR$, where $CF_E$ is case fraction (proportion of exposed cases) and RR relative risk (Miettinen, 1974). Significance of differences between average ages and between average smoking times was determined by the $t$ test (cf. Armitage, 1983). All analyses were performed for two sets of cases and controls. *In the study model A, all the cases and their matched deceased controls were used. In the study model B cases aged under 80 years and their matched living controls were used. Estimates based on study model B are in the text and in Table IV presented in parentheses after the estimates based on study model A.*

### Results

The crude risk ratio for all smokers in the material was 7.3 compared to deceased controls, and 9.0 compared to living controls (Table I). Many (75%) of the youngest deceased controls (<60 y) were smokers, and for this age group a relatively low odds ratio was thus obtained. About 80% of the smoking cases and controls started to smoke before the age of 20 (Table II). For smokers the relative

**Table I** Relative lung cancer risks in smokers

| *Study model* | *Age at diagnosis* | | *Non-smokers* | *Smokers* | $\widehat{OR}$ |
|---|---|---|---|---|---|
| A | <60 | Cases | 11 | 106 | 3.2 |
| | | Controls | 28 | 85 | |
| | 60–69 | Cases | 8 | 170 | 10.2 |
| | | Controls | 57 | 119 | |
| | ≧70 | Cases | 23 | 261 | 8.7 |
| | | Controls | 123 | 160 | |
| | Total | Cases | 42 | 537 | |
| | | Controls | 208 | 364 | |
| | $\widehat{RR}$ (unadjusted) | | (1.0) | 7.3 | |
| | 95% Conf. interval | | | 5.1–10.7 | |
| | $\widehat{RR}$ (adjusted for age) | | (1.0) | 7.3 | |
| | Test for homogeneity ($\chi^2_2$) | | | 5.5 | |
| B | <60 | Cases | 11 | 106 | 5.6 |
| | | Controls | 42 | 72 | |
| | 60–69 | Cases | 8 | 169 | 13.5 |
| | | Controls | 69 | 108 | |
| | ≧70 | Cases | 10 | 146 | 9.1 |
| | | Controls | 60 | 96 | |
| | Total | Cases | 29 | 421 | |
| | | Controls | 171 | 276 | |
| | $\widehat{RR}$ (unadjusted) | | (1.0) | 9.0 | |
| | 95% Conf. interval | | | 5.8–14.2 | |
| | $\widehat{RR}$ (adjusted for age) | | (1.0) | 9.0 | |
| | Test for homogeneity ($\chi^2_2$) | | | 2.7 | |

**Table II** Age at which smoking began

| *Age* | *Cases* | *Deceased controls* | *Living controls* |
|---|---|---|---|
| <15 | 206 | 98 | 77 |
| 16–20 | 261 | 190 | 155 |
| >20 | 70 | 76 | 44 |
| Total | 537 | 364 | 276 |

risk increased successively with smoking time (Figure 1). Smoking for more than 50 years gave about 10 times higher risk than smoking for less than 20 years.

**Figure 1** Relative risks, by smoking years. Clear histogram = study model A. Stippled histogram = study model B.

### *Different types of smokers*

The distribution of different types of smokers are shown in Table III. Pipe smoking was in this series as common as cigarette smoking, both among cases and controls. Cigar smoking on the contrary was very unusual in this material. The relative risk for pure cigarette smokers was 7.0(9.2) and for pipe smokers 6.9(8.1) without regard to quantity of smoking. The average age at diagnosis of lung cancer was significantly higher ($P<0.001$) for the pipe smokers (69.5 y) than for the cigarette smokers

**Table III** Distribution of different types of smoking among cases, deceased controls and living controls

| *Type of smokers* | *Cases* | | *Deceased controls* | | *Living controls* | |
|---|---|---|---|---|---|---|
| | *Number* | *Per cent* | *Number* | *Percent* | *Number* | *Percent* |
| Non-smokers | 42 | 7.3 | 208 | 36.4 | 171 | 38.2 |
| Cigarettes only | 198 | 34.2 | 140 | 24.5 | 108 | 24.2 |
| Pipe only | 198 | 34.2 | 142 | 24.8 | 107 | 23.9 |
| Combination (cigarettes and pipe) | 134 | 23.1 | 75 | 13.1 | 53 | 11.9 |
| Cigars only | 7 | 1.2 | 7 | 1.2 | 8 | 1.8 |

(65.4 y). The pipe smokers also had significantly longer average smoking time (49.1 y) than the cigarette smokers (45.5 y, $P<0.01$). Among both cigarette smokers and pipe smokers the relative lung cancer risk increased with tobacco consumption (Figures 2, 3). The relative risk for individuals smoking more than 25 cigarettes a day was 14.9(33.4) (Figure 2). Heavy pipe smokers (>100 g a week) had a relative risk of 11.1(26.6) while for light pipe smokers (<100 g) this risk was only 4.7(4.3); (Figure 3). Combination smokers (cigarettes and pipe) had a relative risk of 8.9(11.8).

**Figure 2** Relative risks, by cigarettes smoked per day. Clear histogram = study model A. Stippled histogram = study model B.

As in the total group of smokers the relative risk increased with smoking time. It was 2.3(2.4) for smoking less than 30 years and 16.2(15.6) for smoking more than 50 years. In the group with combined cigarette and pipe smoking the intensity was difficult to assess and this group was therefore not further analysed.

### *Effects of smoking cessation*

Among the controls defined as smokers 79(67) were ex-smokers of more than 10 years standing i.e. 22% (24%). The corresponding figures among the cases were 42(26) and 8%(6%). Figure 4 illustrates the effect of smoking cessation in the total material. The relative risk was after 1–5 years of smoking cessation about the same as in current smokers but then gradually decreased, and was after more than 10 years only 2.6(2.3). This reduction was, however, dependent upon the previous smoking time (Figure 4). The decrease of the relative risk in ex-smokers was less pronounced in pipe smokers than in cigarette smokers (Figure 5); in both groups, however, it seemed to be influenced by the previous smoking time.

### *Risk estimates in different types of lung cancer*

High relative risks for smoking were obtained in small cell carcinoma, squamous cell carcinoma and the heterogenous group of large cell anaplastic carcinoma and poorly differentiated carcinoma not further classified. A significantly increased but considerably lower risk was found in the adeno-carcinoma group (Table IV). For small cell and squamous cell carcinomas the risk increased markedly with smoking time (Table V). In study model A, which included all cases regardless of age, it was of interest to compare some findings related to age. The mean age at diagnosis was rather similar (67–68 y) in the 4 mentioned subgroups. Pure cigarette smokers were more common among

**Figure 3** Relative risks, by pipe smoking years and pipe tobacco consumption (g/week). Clear histogram = study model A. Stippled histogram = study model B.

**Table IV** Relative risks for different types of lung cancer

| | | *Smokers* | *Non-smokers* | $\widehat{RR}$ | 95% *Conf. interval* |
|---|---|---|---|---|---|
| Small cell carcinoma | Cases<br>Controls | 145(119)<br>99(68) | 5(2)<br>47(51) | 13.8(44.6) | 5.2–45.6(11.0–385) |
| Adenocarcinoma, alveolar cell carcinoma and bronchiolar carcinoma | Cases<br>Controls | 65(50)<br>49(36) | 16(10)<br>29(22) | 2.4(3.1) | 1.1–5.3(1.2–8.1) |
| Squamous cell carcinoma | Cases<br>Controls | 271(211)<br>169(137) | 14(12)<br>103(76) | 11.8(9.8) | 6.4–23.0(5.0–20.4) |
| Poorly differentiated carcinoma (not further classified) and large cell anaplastic carcinoma | Cases<br>Controls | 39(30)<br>24(19) | 4(3)<br>18(14) | 7.3(7.4) | 2.0–32.5(1.7–43.9) |

**Figure 4** Relative risks, by years of smoking cessation and smoking years before cessation. All smokers. Clear histogram = study model A. Stippled histogram = study model B.

**Table V** Relative risks for different types of lung cancer, by smoking years (study model A)

| | *Small cell carcinoma* | | *Adenocarcinoma, alveolar cell carcinoma and bronchiolar carcinoma* | | *Squamous cell carcinoma* | |
|---|---|---|---|---|---|---|
| *Smoking years* | $\widehat{RR}$ | *95% Confidence interval* | $\widehat{RR}$ | *95% Confidence interval* | $\widehat{RR}$ | *95% Confidence interval* |
| <30 | 3.6 | 1.0–14.3 | 1.8 | 0.6–5.4 | 4.4 | 1.8–10.7 |
| 31–40 | 10.5 | 3.4–38.4 | 1.2 | 0.2–6.0 | 8.4 | 4.0–18.3 |
| 41–50 | 19.6 | 6.5–69.3 | 3.4 | 1.3–9.1 | 13.8 | 6.8–29.1 |
| ≧51 | 25.1 | 8.2–89.0 | 2.5 | 0.9–6.7 | 16.7 | 8.5–34.0 |

the smokers with small cell carcinoma than among the smokers with squamous cell carcinoma (41% *versus* 34%), while the reverse was true for pure pipe smokers (30% *versus* 41%). Only the last difference was significant ($P<0.05$). Cigarette smoking and pipe smoking gave, however, very similar relative risk estimates for these two types of lung cancer.

### Discussion

The main observations in this study agreed with previous reports concerning the major role of smoking as a cause of male lung cancer. The population aetiologic fraction attributable to smoking in the present material was 80%(83%). The relative risks estimated were strikingly similar to those obtained in the above-mentioned Swedish cohort (Cederlöf, 1975). However, these risks were lower than those estimated in the UK and US (Table VI) which may be due to quantitative and qualitative differences in smoking habits.

Most studies have indicated that cigarette smoking is more dangerous than pipe smoking with reference to lung cancer risk (Table VII). However, both the Swedish cohort study and the present investigation gave about the same relative risk for

**Figure 5** Relative risks, by years of smoking cessation and smoking years before cessation. Cigarette (a) and pipe (b) smokers. Clear histogram = study model A. Stippled histogram = study model B.

**Table VI** Reported risk ratios by smoking intensity (cigarettes)

| *Author* | *Type of study* | *Cigarettes per day* | *Risk ratios* | *All cigarette smokers* |
|---|---|---|---|---|
| Doll & Peto (1976) | Cohort | 1–14 | 7.8 | |
| | | 15–24 | 12.7 | 14.0 |
| | | ≧25 | 25.1 | |
| Hammond (1966) | Cohort | 1–9 | 4.6 | |
| | | 10–19 | 8.6 | 9.2 |
| | | 20–39 | 14.7 | |
| | | ≧40 | 18.8 | |
| Cederlöf *et al.* (1975) | Cohort | 1–7 | 2.3 | |
| | | 8–15 | 8.8 | 7.0 |
| | | ≧16 | 13.9 | |
| Present study | Case-control | 1–7 | 2.3(2.3) | |
| | | 8–15 | 7.3(7.0) | 7.0(9.2) |
| | | ≧16 | 10.2(18.2) | |

**Table VII** Reported relative risks of cigarette and pipe smoking

| *Author* | *Type of study* | *Relative risk* | |
|---|---|---|---|
| | | *Cigarette* | *Pipe* |
| Wynder & Graham (1950) | Case-control | 15.7 | 3.6 |
| Doll & Hill (1952) | Case-control | 9.6 | 5.1 |
| Randig (1955) | Case-control | 5.0 | 5.0 |
| Pernu (1960) | Case-control | 9.2 | 4.2 |
| Stocks (1957) | Case-control | 5.0 | 3.1 |
| Cederlöf *et al.* (1975) | Cohort | 7.0 | 7.1 |
| Lubin *et al.* (1984) | Case-control | 9.0 | 2.5 |
| Present study | Case-control | 7.0(9.2) | 6.9(8.1) |

both types of smoking. A similar finding was made in a German study (Randig, 1955). The pipe smokers among the cases in the present study, however, had on average a significantly higher age at diagnosis and more smoking years than the cigarette smoking cases. In a way pipe smoking was thus somewhat less dangerous as longer exposure was required for induction of lung cancer. On the other hand, cohort studies with relatively short observation time probably underestimate the lifetime lung cancer risk of pipe compared to cigarettes.

The decreasing relative risk observed for ex-smokers after more than 5 years is in close agreement with several previous reports (cf. Reif, 1981) and is generally regarded as a strong indicator of a promoting effect of cigarette smoking. The present study furthermore suggested that the reduction of the relative risk in ex-smokers was dependent upon the previous smoking time. In ex-pipe smokers a high relative risk still persisted after 10 years, which might have been due to more irreversible changes caused by the long smoking histories. Another possible explanation could have been differences between the occupational profiles in pipe and cigarette smokers. No indication of an overrepresentation of risk occupations concerning lung cancer, however, was found among the pipe smokers. On the contrary, farmers and forestry workers were over-represented, i.e. groups which in Sweden have lung cancer incidence below the average.

In the present study, the highest relative risks were estimated for small cell and squamous cell carcinoma (Table IV, V). This is in close agreement with most other reports (cf. Surgeon General: Smoking & Health, 1979). An observation of some relevance may be that pipe smoking was more common than cigarette smoking in cases with squamous cell carcinoma, while the reverse was found in cases with small cell carcinoma. Nevertheless the relative risks estimated for pipe and cigarette smoking in the respective types of lung cancer were very similar. This might, however, have been an effect of overmatching. The controls were matched with the cases as regards municipality and the smoking habits had some geographical association, with pipe smoking being more common in the rural municipalities.

As regards the adenocarcinoma group, previous reports are somewhat conflicting. Some early studies showed no or only slight association between smoking and adenocarcinoma (Kreyberg, 1969; Doll & Hill, 1964). Other studies, however, have strongly suggested that smoking also increases the risk for adenocarcinoma (Haenszel *et al.*, 1962; Weiss *et al.*, 1972; Stayner & Wegman, 1983). In the present study, a significantly increased relative risk was estimated for this tumour group but it was considerably lower than for small cell and epidermoid carcinoma.

In the present study, two types of controls were used, living and deceased. For deceased controls, as for the cases, the data were collected through close relatives and from this point of view these two groups were comparable. As smoking is also related to causes of death other than lung cancer, a comparison with deceased controls probably under-estimates the true risk of lung cancer. Living controls, who were matched with the cases according to year of birth and thus had outlived the cases by 2–7 years, may represent a positively selected group conderning disease risk and therefore cause overestimation of the risk. In the present study comparison with living controls as a rule gave higher risk estimates than comparison with deceased controls. It is possible that the estimated relative risks can be regarded as upper (comparison with living controls) and lower (comparison with deceased controls) limits with the true values some-where between. However, ex-smokers among the living controls would less often describe themselves as non-smokers than would surrogate respondents, an effect which would tend to reduce the relative risk. Thus it cannot be excluded that even the relative risks obtained by comparison with living controls actually represented an underestimation.

This work was supported by the Swedish Cancer Society and by the Swedish Work Environment Fund. We are deeply indepted to Miss Ulla Jonsson and Mrs Monica Johnsson for their skilful help with the questionnaires and Mr Claes Olsson for his valuable help with the telephone interviews.

### References

ARMITAGE, P. (1983). *Statistical Methods in Medical Research*. Blackwell Scientific Publications: London.

CEDERLÖF, R., FRIBERG, L., HRUBEC, Z. & LORICH, U. (1975). The relationship of smoking and some social covariables to mortality and cancer morbidity. A ten year follow-up in a probability sample of 55,000 subjects age 18–69. Parts 1 and 2. Department of Environmental Hygiene, Karolinska Institute: Stockholm.

DAMBER, L. & LARSSON, L.G. (1982). Combined effects of mining and smoking in the causation of lung carcinoma. A case-control study in northern Sweden. *Acta Radiol. Oncol.*, **21**, 305.

DAMBER, L. & LARSSON, L.G. (1985). Professional driving, smoking, and lung cancer: A case-referent study. *Br. J. Industr. Med.*, **42**, 246.

DOLL, R. & HILL, A.B. (1950). Smoking and carcinoma of the lung: Preliminary report. *Br. Med. J.*, **2**, 739.

DOLL, R. & HILL, A.B. (1952). A study of the aetiology of carcinoma of the lung. *Br. Med. J.*, **2**, 1271.

DOLL, R. & HILL, A.B. (1964). Mortality in relation to smoking: Ten years' observations of British doctors. *Br. Med. J.*, **1**, 1399, 1460.

DOLL, R. & PETO, R. (1976). Mortality in relation to smoking: 20 years' observations on male British doctors. *Br. Med. J.*, **2**, 1525.

HAENSZEL, W., LOVELAND, D. & SIRKEN, M. (1962). Lung cancer mortality as related to residence and smoking histories. I. White males. *J. Natl Cancer Inst.*, **28**, 947.

HAMMOND, E.C. (1966). Smoking in relation to the death rates of one million men and women. *Natl Cancer Inst., Monogr.*, **19**, 127.

KREYBERG, L. (1969). *Aetiology of lung cancer: A morphological, epidemiological, and experimental analysis*. Universitetsförlaget: Oslo.

LEVIN, M.L., GOLDSTEIN, H. & GERHARDT, P.R. (1950). Cancer and tobacco smoking: Preliminary report. *JAMA*, **143**, 336.

LUBIN, J.B., RICHTER, B.S. & BLOT, W.J. (1984). Lung cancer risk with cigar and pipe use. *J. Natl Cancer Inst.*, **73**, 377.

MANTEL, N. & HAENSZEL, W. (1959). Statistical aspects of the analysis of data from retrospective studies of disease. *J. Natl Cancer Inst.*, **22**, 719.

MIETTINEN, O.S. (1974). Proportion of disease caused or prevented by a given exposure, trait or intervention. *Am. J. Epidemiol.*, **99**, 325.

MIETTINEN, O.S. (1975). Principles of epidemiologic research. Cited: *Epidemiologic analysis with a programmable calculator* (1978), Rothman, K.J. & Boice, J.D. (eds) p. 6. US Dept. of Health, Education and Welfare: Washington DC.

PERNU, J. (1960). An epidemiological study on cancer of the digestive organs and respiratory system. A study based on 7,078 cases. *Ann. Med. Interne Fenniae*, **49**(Suppl. 33), 1.

RANDIG, K. (1955). Zür Ätiologie des Lungenkrebses. *Dtsch. Med. Wochenschr.*, **80**, 718.

REIF, A.E. (1981). Effect of cigarette smoking on susceptibility to lung cancer. *Oncology*, **38**, 76.

SMOKING AND HEALTH: A report of the Surgeon General. (1979). *US Dept. of Health, Education and Welfare. Office of the Assistant Secretary for Health, Office on Smoking and Health. DHEW publication* No. (PHS) 79-50066, p. 1136.

STAYNER, L.T. & WEGMAN, D.H. (1983). Smoking, occupation and histopathology of lung cancer: A case-control study with the use of the third national cancer survey. *J. Natl Cancer Inst.*, **70**, 421.

STOCKS, P. (1957). Cancer incidence in North Wales and Liverpool region in relation to habits and environment. *Ann. Rep. Brit. Empire Cancer Campaign*. (Suppl. to Part II). p. 156.

THOMAS, D.G. (1971). Exact confidence limits for the odds ratio in a $2 \times 2$ table. *Appl. Stat.*, **20**, 105.

WEISS, W., BOUCOT, K., SEIDMAN, H. & CARNAHAN, W. (1972). Risk of lung cancer according to histologic type and cigarette dosage. *JAMA*, **222**, 799.

WYNDER, E.L. & GRAHAM, E.A. (1950). Tobacco smoking as a possible etiologic factor in bronchiogenic carcinoma. *JAMA*, **143**, 329.

WYNDER, E.L. & GOODMAN, M.T. (1983). Smoking and lung cancer: Some unresolved issues. *Epidemiol. Rev.*, **5**, 177.